# Correlation of Intraoperative 5-ALA-Induced Fluorescence Intensity and Preoperative ^11^C-Methionine PET Uptake in Glioma Surgery

**DOI:** 10.3390/cancers14061449

**Published:** 2022-03-11

**Authors:** Kazuhide Shimizu, Kaoru Tamura, Shoko Hara, Motoki Inaji, Yoji Tanaka, Daisuke Kobayashi, Takashi Sugawara, Hiroaki Wakimoto, Tadashi Nariai, Kenji Ishii, Ichiro Sakuma, Taketoshi Maehara

**Affiliations:** 1 Department of Neurosurgery, Tokyo Medical and Dental University, 1-5-45 Yushima, Bunkyo-ku, Tokyo 113-8519, Japan; tamura.nsrg@tmd.ac.jp (K.T.); shara.nsrg@tmd.ac.jp (S.H.); inaji.nsrg@tmd.ac.jp (M.I.); tanaka.nsrg@tmd.ac.jp (Y.T.); sugawara.nsrg@tmd.ac.jp (T.S.); nariai.nsrg@tmd.ac.jp (T.N.); maehara.nsrg@tmd.ac.jp (T.M.); 2 Department of Neurosurgery, Massachusetts General Hospital, Harvard Medical School, 185 Cambridge St, Boston, MA 02114, USA; hwakimoto@mgh.harvard.edu; 3 Research Team for Neuroimaging, Tokyo Metropolitan Institute of Gerontology, 35-2 Sakaecho, Itabashi-ku, Tokyo 173-0015, Japan; ishii@pet.tmig.or.jp; 4 Department of Human Pathology, Tokyo Medical and Dental University, 1-5-45, Yushima, Bunkyo-ku, Tokyo 113-8519, Japan; d-koba.pth1@tmd.ac.jp; 5 Graduate School of Engineering, The University of Tokyo, 7-3-1 Hongo, Bunkyo-ku, Tokyo 113-8654, Japan; sakuma@bmpe.t.u-tokyo.ac.jp

**Keywords:** 5-ALA, 5-aminolevulinic acid, MET-PET, glioma, glioblastoma

## Abstract

**Simple Summary:**

In malignant brain tumor surgery, precise identification of the tumor is essential. 5-Aminolevulinic acid (5-ALA) labels tumor cells with red fluorescence to facilitate tumor resection. On the other hand, the nuclear medicine imaging technique, positron emission tomography with ^11^C-methionine (MET-PET), can delineate tumors precisely but is not widely available. This study aimed to determine the correlation between intraoperative 5-ALA-induced fluorescence and preoperative MET-PET signals of gliomas. We quantitatively measured the fluorescence intensity from tumor samples and calculated the MET-PET uptake by the tumor. Our study showed that strong tumor fluorescence correlated with high MET-PET uptake and cellular proliferation. Our findings might be valuable to rapidly provide information on tumor biology at the time of surgery in circumstances where MET-PET is inaccessible.

**Abstract:**

Background: 5-Aminolevulinic acid (5-ALA) is widely employed to assist fluorescence-guided surgery for malignant brain tumors. Positron emission tomography with ^11^C-methionine (MET-PET) represents the activity of brain tumors with precise boundaries but is not readily available. We hypothesized that quantitative 5-ALA-induced fluorescence intensity might correlate with MET-PET uptake in gliomas. Methods: Adult patients with supratentorial astrocytic gliomas who underwent preoperative MET-PET and surgical tumor resection using 5-ALA were enrolled in this prospective study. The regional tumor uptake of MET-PET was expressed as the ratio of standardized uptake volume max to that of the normal contralateral frontal lobe. A spectrometric fluorescence detection system measured tumor specimens’ ex vivo fluorescence intensity at 635 nm. Ki-67 index and IDH mutation status were assessed by histopathological analysis. Use of an antiepileptic drug (AED) and contrast enhancement pattern on MRI were also investigated. Results: Thirty-two patients, mostly with Glioblastoma IDH wild type (46.9%) and anaplastic astrocytoma IDH mutant (21.9%), were analyzed. When the fluorescence intensity was ranked into four groups, the strongest fluorescence group exhibited the highest mean MET-PET uptake and Ki-67 index values. When rearranged into fluorescence Visible or Non-visible groups, the Visible group had significantly higher MET-PET uptake and Ki-67 index compared to the Non-visible group. Contrast enhancement on MRI and IDH wild type tumors were more frequent among the Visible group. AED use did not correlate with 5-ALA-induced fluorescence intensity. Conclusions: In astrocytic glioma surgery, visible 5-ALA-induced fluorescence correlated with high MET-PET uptake, along with a high Ki-67 index.

## 1. Introduction

The most common primary malignant brain tumor in adults is glioblastoma (GBM). This cancer, along with other tumor types of the same lineage (collectively adult malignant gliomas), has a dismal prognosis despite the current intensive treatment strategies [1,2]. GBM, in particular, has an infiltrative nature that makes complete surgical removal challenging and is resistant to radiation therapy and chemotherapies. Still, aggressive or extensive surgical removal has been associated with a better prognosis for GBM and other malignant gliomas [3,4].

To facilitate maximal safe reduction of the tumor, fluorescence-guided surgery (FGS) has become a widely employed technique to identify the presence of tumor cells intraoperatively [5,6,7]. In FGS, tumor-specific accumulation of a photosensitizer is necessary to label the tumor with fluorescence. Following the administration of the photosensitizer (or its precursor), direct illumination of excitation light with a specific wavelength invokes fluorescence emission to reveal the residual tumor’s presence intraoperatively. This technique is called photodynamic diagnosis (PDD) [8]. Among many potential photosensitizers, 5-Aminolevulinic acid (5-ALA) is most intensively studied in FGS for malignant brain tumors [9,10,11,12]. 5-ALA is one of the amino acids and a natural precursor in heme biosynthesis and hence is notably safe [10,13]. When 5-ALA is administered orally, it gets metabolized into the fluorescent molecule Protoporphyrin IX (PpIX) that accumulates specifically in tumor cells by various mechanisms, including a lack of enzymes that convert PpIX to heme and decreased activity of transporters [13,14,15,16,17]. In addition, 5-ALA is known to cross the blood–brain barrier (BBB) [18], and 5-ALA-induced fluorescence in brain tumors correlates with the malignancy of the tumor cells [19,20,21,22] and has >80% sensitivity and specificity [23]. Consequently, FGS with 5-ALA has been approved in many countries and proved to enhance the gross total resection of GBM [24,25].

Preoperatively, tumor localization and its boundary must be vigorously investigated predominantly by imaging studies to assist brain tumor surgery. Magnetic resonance imaging (MRI) with gadolinium contrast enhancement is the most accepted technique for diagnosing this actively proliferating tumor. In general, GBM exhibits solid enhancement on the rim and minor or no enhancement in the necrotic tissue core (ring enhancement) due to the BBB breakdown in and around the tumor. More recently, positron emission tomography (PET) imaging with metabolic tracers, especially amino acid tracers such as ^11^C-methionine (MET), has been reported to delineate brain tumors with better sensitivity [26] in the preoperative diagnosis and postoperative follow-up [27]. Furthermore, because MET accumulates through amino acid transporters [28] and reflects the active metabolism of the cell [29], MET-PET uptake can occur without the BBB breakdown, allowing MET-PET to properly represent the activity or malignancy of the brain tumor [30] with more precise boundaries [31]. Despite the potential advantages of MET-PET in diagnosing GBM, however, its availability is limited since MET-PET requires special facilities such as a cyclotron [32].

Given these backgrounds, we hypothesized that quantitative 5-ALA-induced fluorescence intensity might correlate with MET-PET uptake as both parameters reflect the tumor cell malignancy. Thus, the purpose of this study is to elucidate the relationship between preoperative MET-PET uptake and intraoperative 5-ALA-induced fluorescence intensity quantified from ex vivo tumor specimens, adding a new axis to the 5-ALA-induced fluorescence in glioma surgery.

## 2. Materials and Methods

### 2.1. Patient Cohort

The patients were enrolled in this prospective study at the Department of Neurosurgery, Tokyo Medical and Dental University, from May 2016 to August 2019. The inclusion criteria were age >20, a diagnosis of supratentorial malignant glioma (newly diagnosed or recurrent), eligibility for surgical resection of the tumor using 5-ALA, and availability of the MET-PET data taken prior to the surgery. Biopsy cases were excluded. Because oligodendrogliomas are known to have higher MET-PET uptake relative to astrocytomas [30], only astrocytic tumors (astrocytomas and GBM) were included in the final analysis. Other clinical data that might affect the presence of 5-ALA-induced fluorescence, such as the use of an antiepileptic drug (AED) and the pattern of Gd-enhancement in the preoperative MRI were also collected.

### 2.2. MET-PET Scan

Positron emission tomography measurements were determined using a PET scanner (Headtome IV or V, Shimadzu, Kyoto, Japan) and presented as the equilibrated radioactivity 20 min after intravenous injection of MET (250–350 MBq), as described previously [33]. The transmission data were acquired for each patient with a rotating germanium-68 rod source for attenuation correction. The regional uptake of MET-PET was expressed as the standardized uptake volume (SUV) ((tissue activity [Bq]/tissue volume [ml])/(injected radioisotope activity [Bq]/bodyweight [g])), and the tracer uptake by the tumor was expressed as the ratio of SUV max of the lesion to that of the normal contralateral frontal lobe (lesion/normal region ratio: L max/N ratio (SUV max of the lesion/SUV max of the normal region)). To determine the SUV of the lesion, the regions of interest were manually placed.

### 2.3. Tissue Sampling and Fluorescence Measurement

The patients received an oral administration of 5-ALA (Alabel Oral, Nobelpharma Co., Ltd., Tokyo, Japan) at 20 mg/kg body weight three hours before elective craniotomy as part of the routine procedures for fluorescence-guided resection of gliomas. Tumor removal was conducted using standard neurosurgical procedures. Tumor samples were obtained from a MET uptake-high region of the tumor according to the image-navigation system using the preoperative images, including Gd-enhanced MRI and MET-PET [34]. Preoperative MET-PET images were fused to MRI images during neuronavigation whenever possible. Otherwise, the MET-PET images were displayed in the operating room for the surgeons to review at any time. 

The ex vivo fluorescence intensity from the tumor specimens was measured immediately after collecting the sample in the operating room. As previously described [35], we used a custom-made fluorescence detection system consisting of a semiconductor laser with 400 nm wavelength, an optic fiber bundle (probe) connected to a cooled CCD array spectrometer (16-bit resolution) for the excitation and the quantitative detection of the fluorescence, and a 3D-printed box to fix the distance between the tumor sample and the optic fiber probe to minimize the deviation of the measured fluorescence intensity. We chose a 400 nm laser due to commercial availability after confirming that this wavelength is included in the excitation spectrum [36]. First, tumor samples were briefly pre-scanned with the 400 nm laser and observed through an optical filter (Kenko Y2 Professional Optical Filter, Kenko Tokina Co., Ltd., Tokyo, Japan) to confirm the presence of fluorescence, if any. Then, the samples were cut into pieces of approximately 10 mm to fit the detection system. The fluorescence intensity was measured at the 635 nm peak by subtracting the baseline at 500 nm.

### 2.4. Histopathological Analysis

All tumor specimens were subject to histopathological analysis by neuropathologists who were blinded to the presence of the fluorescence. Hematoxylin and eosin (H&E) staining and immunohistochemistry (Ki-67 as the proliferating index, IDH1-R132H for detecting isocitrate dehydrogenase mutation) were used as part of routine procedures to make final diagnosis according to 2016 WHO Classification of Tumors of the Central Nervous System. 

### 2.5. Statistical Analysis

Fluorescence intensity was compared by non-parametric analysis. The Unpaired *t* test and one way ANOVA test compared two and multiple groups, respectively. In addition, the Pearson Chi-square test was used to test the frequency of the two variables. All *p*-values were two-sided, and *p*-values less than 0.05 were considered statistically significant. All statistical analyses were conducted using JMP version 14.2.0 (SAS Institute Inc., Cary, NC, USA).

## 3. Results

A total of 52 adult patients with supratentorial tumors suspected of malignant glioma underwent surgical resection using 5-ALA, and 32 patients were included in the final analysis after excluding those without adequate MET-PET data or those diagnosed other than astrocytic tumors (Figure S1). 

Table 1 summarizes the patient characteristics who were included in the analysis. The median age was 53 (range: 23–81), with 14 females (43.8%) and 18 males (56.3%). The pathological diagnoses were GBM, IDH wild type, 15 (46.9%); GBM, IDH mutant, 2 (6.3%); GBM, nos (not otherwise specified), 1 (3.1%); Anaplastic astrocytoma, IDH wild type, 3 (9.4%); Anaplastic astrocytoma, IDH mutant, 7 (21.9%); Diffuse astrocytoma, IDH wild type, 1 (3.1%); Diffuse astrocytoma, IDH mutant, 3 (9.4%). Newly diagnosed tumors accounted for 23 cases (71.9%).

**Figure 1:**
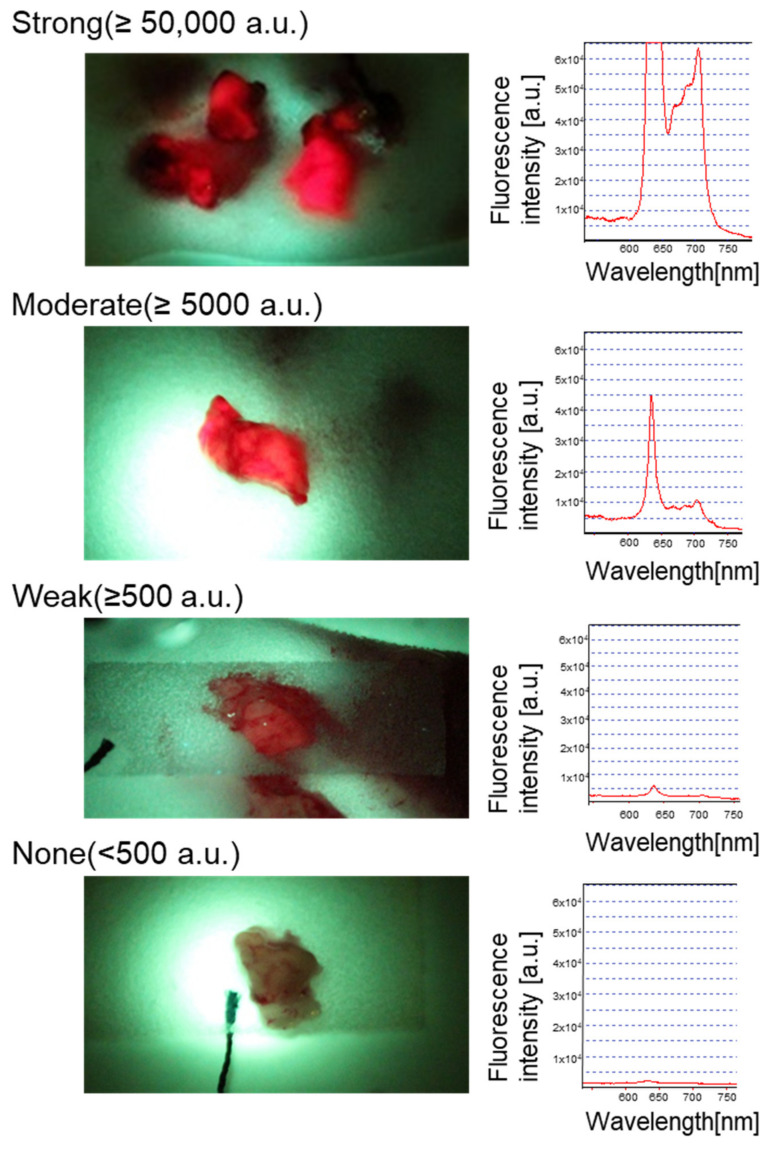
Quantification of intraoperative 5-ALA-induced fluorescence intensity from resected tumor samples.

Figure 1 shows examples of quantification of the intraoperative 5-ALA-induced fluorescence intensity from resected tumor specimens. The 5-ALA-induced red fluorescence from each sample is displayed with the corresponding spectrum (excitation laser peak at 400 nm, fluorescence peak at 635 nm). Because 5-ALA-induced fluorescence is remarkably bright when present, the intensity peak exceeded the detection system’s dynamic range (16-bit) in 20 samples (as exemplified by Figure 1 top), reflecting the feature of our cohort that is predominantly high-grade disease (WHO grade III and IV) (Table 1). According to the classic law of human perception of the stimuli (Weber–Fechner law), the internal subjective sense of magnitude is proportional to the logarithm of stimulus intensity [37]. Therefore, the tumor samples were ranked into four groups with ten-fold thresholds within the dynamic range (None, <500 a.u. (N = 4); Weak, 500–4999 a.u. (N = 3); Moderate, 5000–49,999 a.u. (N = 3); Strong, >50,000 a.u. (N = 22)) to facilitate comparison.

Fresh surgical specimens from the tumor indicated by the navigation system were collected and subject to fluorescence measurement in the operating room. Samples were cut into pieces approximately 10 mm in diameter before scanning the fluorescence spectrum. A quantitative spectroscopic fluorescence excitation and detection system was used as previously described [35], with a minor modification (the peak fluorescence intensity was calculated at 635 nm). The fluorescence intensity fell under four groups by ten-fold (None, <500 a.u.; Weak, 500–4999 a.u.; Moderate, 5000–49,999 a.u.; Strong, >50,000 a.u.) to facilitate the comparison. The picture and the spectrum of each row represent the fluorescence of each group. Note that the strong signal coming from the tumor shown at the top made the fluorescence intensity peak exceed the detection system’s dynamic range (16-bit).

**Figure 2:**
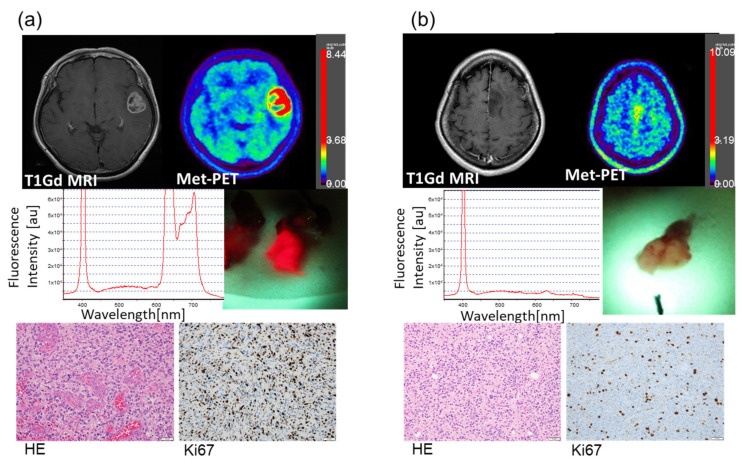
Illustrative cases. (**a**) A 67-year-old female with a newly diagnosed tumor in the left temporal lobe. Preoperative imaging showed ring enhancement on T1Gd MRI and high uptake on MET-PET. The SUV max (T/N ratio) of the MET-PET scan was 4.66. Bright red 5-ALA-induced fluorescence from the surgical specimen was captured and the intensity was detected by the quantification system. The peak at 635 nm was scaled out of the measurement range. There is a second peak of around 700 nm. This peak became apparent due to the high signals and was consistent with previous studies in which this second peak was noted [36]. Histopathological analysis revealed high cellularity with microvascular proliferation in hematoxylin and eosin-stained section. Immunohistochemistry with Ki-67 labeling showed abundant proliferating tumor cells with 43.4% positivity. The final diagnosis was Glioblastoma, IDH wild type. (**b**) A 44-year-old female with a newly diagnosed tumor in the left frontal lobe. In the preoperative imaging, the tumor showed no enhancement on MRI, while the SUV max (T/N ratio) of the MET-PET scan was only mildly elevated (2.57). Repeated scanning of the tumor specimens failed to detect 5-ALA-induced fluorescence. Histopathology revealed densely proliferating tumor cells with Ki-67 index of 14.0%, without evidence of microvascular proliferation or necrosis. The final diagnosis was Anaplastic astrocytoma, IDH mutant.

Two illustrative cases are shown in Figure 2. Case number 4 (Figure 2a) was a 67-year-old female with a newly diagnosed GBM IDH wild type in the left temporal lobe. Along with the ring enhancement on T1-Gd MRI, MET-uptake (T/N ratio) was elevated to 4.66. In addition, bright red fluorescence was detected from the intraoperative tumor specimen. The corresponding fluorescence spectrum showed a markedly heightened intensity peak at 635 nm, which exceeded the dynamic range of the measurement system. Histopathological analysis revealed abundant proliferating tumor cells (Ki-67 43.4%) with microvascular proliferation, consistent with the diagnosis of GBM, IDH wild type. Case number 22 (Figure 2b) was a 44-year-old female with a newly diagnosed anaplastic astrocytoma in the left frontal lobe. Without enhancement of the tumor on T1-Gd MRI, MET-PET uptake was slightly elevated to 2.57. Despite multiple scanning of the tumor specimens, no evidence for 5-ALA-induced fluorescence was found. The tumor cells were dense on H and E stained sections without typical features for GBM, such as microvascular proliferation or necrosis, and the Ki67 index was 14.0%. The final diagnosis was anaplastic astrocytoma, IDH mutant. 

**Figure 3:**
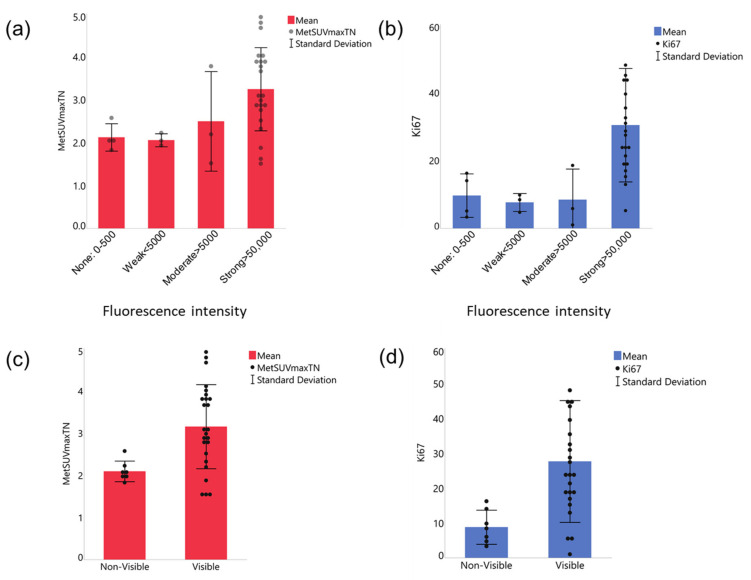
Relationships between MET-PET uptake and Ki-67 index versus quantified 5-ALA-induced fluorescence intensity. (**a**,**b**) Comparison between 4 fluorescence intensity groups. Both MET-PET (**a**) and Ki-67 index (**b**) were the highest in the strong fluorescence group. The one way ANOVA test revealed that the means of the 4 groups are not equal for both Ki-67 index and MET-PET uptake. However, the Tukey method’s posthoc analysis found no statistical significance in any pair. (**c**,**d**) Comparison of two fluorescence intensity groups rearranged based on signal visibility, Visible (including Strong and Moderate groups) or Non-visible (Weak and None), for MET-PET uptake (**c**) and Ki-67 index (**d**). Unpaired *t* tests were significant for MET-PET uptake (**c**, *p* < 0.001) and Ki-67 (**d**, 0.0091). The data are shown by means (solid bars) and standard deviation (error bars), with each dot representing a tumor.

We next compared the four tumor groups of varying intraoperatively measured fluorescence intensities for the levels of preoperative MET-PET uptake (Figure 3a). We also compared the four fluorescence groups for differences in the Ki-67 index of the tumors. (Figure 3b). The Strong fluorescence group exhibited the highest mean MET-PET uptake and Ki-67 index values, compared with the other groups. The one way ANOVA test rejected the null hypothesis that all means are equal. However, the post hoc analysis failed to detect significant differences between the two chosen groups. 

We next rearranged the tumors into two groups: Visible (including Strong and Moderate groups, N = 25) or not: Non-visible (Weak and None, N = 7), because we noted that the fluorescence from ex vivo tumors was obviously visible when the intensity is above 5000 a.u. Our further analysis of the relationship between the MET-PET uptake or Ki-67 index and fluorescence intensity showed that the Visible group had significantly higher MET-PET uptake (Figure 3c) and Ki-67 index (Figure 3d) compared to the Non-visible group (*p* < 0.001 for MET-PET uptake and *p* = 0.0091 for Ki-67 index, Unpaired *t* tests). 

**Figure 4:**
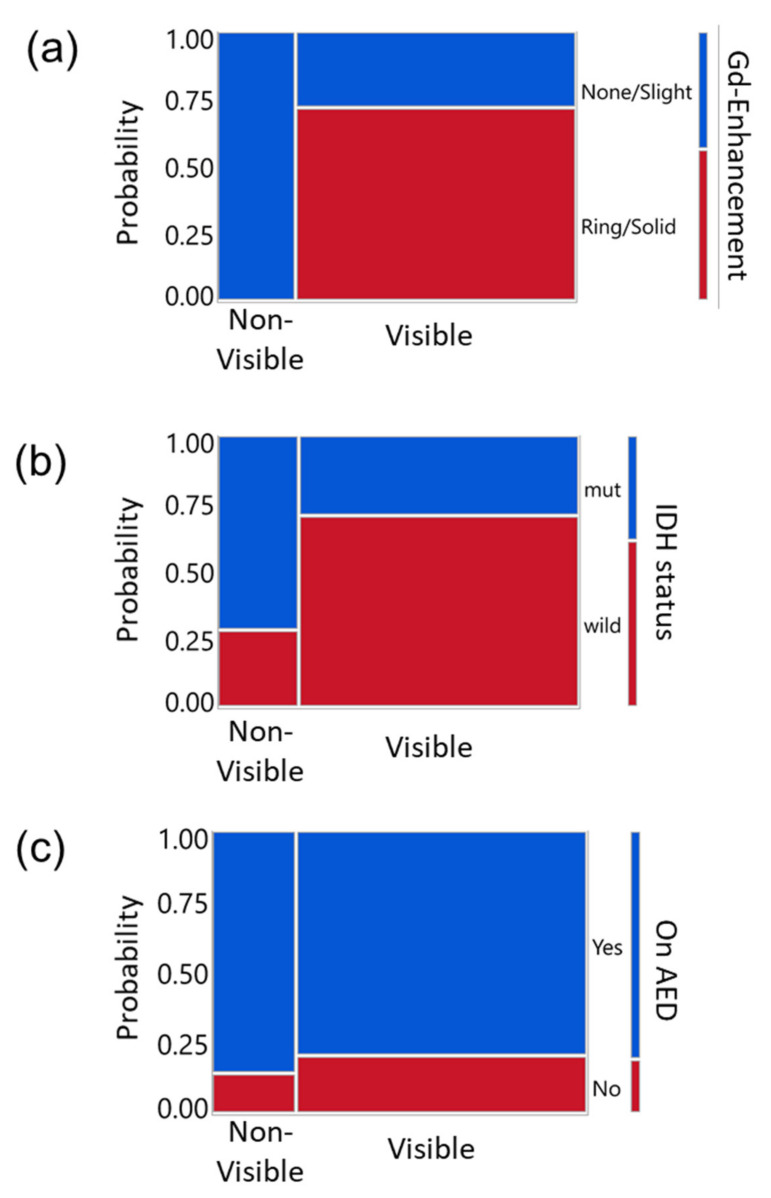
Relationships between other clinical parameters versus quantified 5-ALA-induced fluorescence intensity in mosaic plots. Y-axis indicates the probability and the color indicates each group to be compared with the visibility of the fluorescence (Visible or Non-visible). Legends are on the right side of each panel. (**a**) Gd enhancement of tumor on the MRI, (**b**) IDH status, and (**c**) use of AED versus 5-ALA induced fluorescence intensity classified into two groups; Visible (including Strong and Moderate groups) or Non-visible (Weak and None). Pearson Chi-Square test yielded significance for the enhancement (**a**, *p* = 0.0007) and IDH status (**b**, *p* = 0.0434) but not for AED use (**c**, *p* = 0.6996).

Lastly, we investigated the association between other parameters (contrast enhancement on the preoperative MRI, IDH status, and AED) and tumor 5-ALA fluorescence using mosaic plots and Pearson Chi-square test (Figure 4). Ring or solid enhancement was more frequently seen in cases with strong (visible) fluorescence; all the Non-visible tumors showed none or slight enhancement on MRI (Figure 4a, *p* = 0.0007). IDH wild type tumors were more common in the subjects with strong fluorescence than non-fluorescent tumors; conversely, IDH-mutant tumors were more frequent in the non-fluorescent tumors than fluorescent tumors (Figure 4b, *p* = 0.0434). In contrast, AED use did not correlate with the intensity of 5-ALA-induced fluorescence (Figure 4c, *p* = 0.6996).

## 4. Discussion

There are only a few reports regarding combining 5-ALA and MET-PET. 5-ALA-induced fluorescence and MET-PET correlated to cell density or Ki-67 index but did not correlate with each other [26,38]. MET-PET is more sensitive in detecting tumors than 5-ALA [39] and the methionine uptake is known to correlate with cell proliferation [40]. However, one of the considerable concerns about evaluating 5-ALA-induced fluorescence is that the fluorescence is often only subjectively judged as “positive” or “negative”. Here, for the first time, we report a direct quantitative correlation between MET-PET uptake and 5-ALA-induced fluorescence intensity. Our study incorporated an ex vivo fluorescence measurement technique based on spectroscopy and categorized the fluorescence intensity quantitatively, allowing an objective comparison with MET-PET data. The results demonstrated that MET-PET uptake was significantly high in tumor specimens with quantitatively confirmed 5-ALA-induced fluorescence, suggesting a correlation between those factors in astrocytic tumors. Furthermore, a cell proliferating index, Ki-67, was significantly increased in the fluorescent specimens. Regarding Ki-67, a previous report compared the Ki-67 index with quantified PpIX concentration (not the fluorescence) from surgical specimens and yielded similar findings to ours [19]. As expected, visible fluorescence correlated with the contrast enhancement on the preoperative MRI and IDH status (wild type). These results can be interpreted as 5-ALA-induced fluorescence being reflective of the tumor’s aggressiveness [22], supporting the reliability of the fluorescence detection system in this study. Although previous reports suggested that the use of AED could negatively affect the generation of 5-ALA-induced fluorescence [41,42,43], our investigation did not show a difference in detected fluorescence intensity according to AED use. This might be because the AEDs used in our study were mainly levetiracetam, while the previous reports included more phenytoin and valproic acid or low-grade glioma cases. 

The mechanism behind the correlation between ^11^C-methionine and 5-ALA (or its metabolite, PpIX) has not been understood. The expression of L-Type amino acid transporter 1 (LAT1: SLC7A5) in human glioma correlates with MET uptake [44]. On the other hand, 5-ALA uptake occurs through cell membrane-embedded active transporters, including peptide transporters (PEPT1, PEPT2), Proton-coupled amino acid transporter 1 (PAT1: SLC36A1), and Na^+^- and Cl^−^-dependent taurine transporter (TAUT: SLC6A6) [45,46,47]. Then, PpIX is eliminated via ATP-binding cassette transporter ABCG2 [48]. Although some of these transporters form an SLC gene superfamily, there is no direct evidence that these transporters for methionine uptake and 5-ALA uptake have cross-talks, to the best of our knowledge. Further research is needed to elucidate the mechanisms that underlie the relationship between the uptake of methionine and 5-ALA. 

A limitation of this study is that the location of the tumor specimen might not have been precisely matched with the preoperative MET-PET images due to the brain shift that occurred during surgery. Therefore, when available, we utilized the specimen exhibiting the strongest fluorescence to correlate with the SUVmax of the MET-PET. Secondly, because of the limited dynamic range of the spectrometer relative to the marked brightness of 5-ALA-induced fluorescence in some cases, our fluorescence intensity data could not be used as a continuous value. Consequently, the data points were distributed somewhat unevenly among the fluorescence intensity groups, making statistical analysis challenging. On another note, the MET-PET uptake was calculated as the SUV max of the lesion over the SUV max of the normal region. Accordingly, the calculated values may look smaller compared with reports in which the SUV max of the lesion is divided by the SUV mean of the normal region [49]. Nevertheless, applying the same method throughout the analysis across the cohort makes this study’s MET-PET uptake data valid. 

Together, the present study demonstrated a correlation between the preoperative MET-PET uptake and Ki-67 index versus intraoperative ex vivo fluorescence intensity in astrocytic glioma surgery. The visible 5-ALA-induced fluorescence may suggest the locus of high MET-PET high uptake. Given the broad clinical availability of the 5-ALA technique, our findings might be valuable to provide information on tumor biology rapidly (e.g., aggressiveness) at the time of surgery in circumstances where MET-PET is inaccessible. Additionally, intraoperative quantitative measurement of excised tumors can inform the surgeon of the presence of viable tumors cells at the resection margins and assist a decision-making process towards maximizing the extent of resection during astrocytic brain tumor surgery.

## 5. Conclusions

In astrocytic glioma surgery, visible 5-ALA-induced fluorescence correlates with high MET-PET uptake, along with a high Ki-67 index. This quantitative, biological insight might add a new axis to the utilities of fluorescence-guided brain tumor surgery using 5-ALA. 

## Figures and Tables

**Table 1:** Patient Characteristics.

Case Number	Age (Years)	Sex	Localization	Histology	WHO Grade	Tumor Status	IDH-1	Ki67	Gd Enhancement	AED	Fluorescence Intesnsity	Met SUV		
												T/N	Tumor	Normal
1	54	M	Parietal	GBM	IV	Recurrent	Wild type	23.5	Ring/Solid	LEV	Strong	2.83	5.72	2.02
2	78	F	Temporal	GBM	IV	New	Wild type	18.9	Ring/Solid	None	Strong	3.82	4.96	1.3
3	59	M	Temporal	GBM	IV	New	Wild type	27.4	Ring/Solid	LEV	Strong	4.78	6.84	1.43
4	67	F	Temporal	GBM	IV	New	Wild type	43.4	Ring/Solid	LEV	Strong	4.66	8.44	1.81
5	78	M	Frontal	GBM	IV	New	Wild type	82.8	Ring/Solid	LEV	Strong	3.05	4.48	1.47
6	37	F	Frontal	GBM	IV	Recurrent	Mutant	35.4	Ring/Solid	LEV	Strong	2.51	6.7	2.67
7	65	M	Temporal	GBM	IV	New	Wild type	18.9	Ring/Solid	LEV	Strong	3.65	4.02	1.1
8	67	F	Temporal	GBM	IV	Recurrent	Wild type	43.8	Ring/Solid	None	Strong	1.51	2.71	1.8
9	53	M	Temporal	GBM	IV	New	Wild type	30.9	Ring/Solid	LEV	Strong	2.95	4.04	1.37
10	72	F	Frontal	GBM	IV	New	Wild type	24.2	Ring/Solid	NA	Strong	4	8.15	2.04
11	38	F	Frontal	GBM	IV	Recurrent	Mutant	48	Ring/Solid	LEV	Strong	4.04	9.65	2.39
12	70	M	Temporal	GBM	IV	New	Wild type	16.9	Ring/Solid	LEV	Strong	4.91	7.42	1.51
13	26	M	Parietal	GBM	IV	Recurrent	Unknown	NA	Ring/Solid	None	Strong	2.8	4.79	1.71
14	72	F	Parietal	GBM	IV	New	Wild type	39.5	None/Slight	None	Strong	1.6	3.27	2.04
15	77	M	Frontal	GBM	IV	New	Wild type	23.6	Ring/Solid	Others	Strong	3.82	5.46	1.43
16	81	F	Frontal	GBM	IV	New	Wild type	32.5	Ring/Solid	LEV	Strong	2.75	5.67	2.06
17	69	M	Frontal	GBM	IV	New	Wild type	45	Ring/Solid	LEV	Strong	3.66	8.09	2.21
18	46	M	Temporal	GBM	IV	New	Wild type	28.6	Ring/Solid	LEV	Strong	3.84	6.45	1.68
19	52	F	Temporal	AA	III	New	Mutant	9.8	None/Slight	None	Weak	1.93	3.24	1.68
20	54	M	Parietal	AA	III	Recurrent	Mutant	4.7	None/Slight	LEV	Weak	2.22	1.71	0.77
21	23	M	Temporal	AA	III	New	Wild type	16.2	None/Slight	LEV	None	1.82	2.35	1.29
22	44	F	Frontal	AA	III	New	Mutant	14	None/Slight	LEV	None	2.57	4.65	1.81
23	23	F	Frontal	AA	III	New	Wild type	8.4	None/Slight	LEV	Weak	2.01	2.87	1.43
24	40	F	Frontal	AA	III	New	Wild type	18.5	Ring/Solid	LEV	Moderate	3.77	7.43	1.97
25	58	M	Frontal	AA	III	New	Mutant	5.2	None/Slight	LEV	Strong	1.87	3.99	2.13
26	33	M	Insula	AA	III	Recurrent	Mutant	21.3	None/Slight	LEV	Strong	2.84	5.4	1.9
27	33	M	Insula	AA	III	Recurrent	Mutant	15.2	None/Slight	LEV	Strong	3.04	7.23	2.38
28	43	M	Frontal	AA	III	New	Mutant	12.9	None/Slight	LEV	Strong	2.32	3.78	1.63
29	31	M	Insula	DA	II	New	Mutant	3.3	None/Slight	LEV	None	2.07	3.72	1.8
30	77	F	Temporal	DA	II	New	Wild type	1	None/Slight	None	Moderate	2.19	3.96	1.81
31	37	F	Insula	DA	II	Recurrent	Mutant	5.8	None/Slight	LEV	Moderate	1.52	2.47	1.63
32	42	M	Temporal	DA	II	New	Mutant	5.1	None/Slight	LEV	None	2.02	4.96	2.46

AA, Anaplastic Astrocytoma; DA, Diffuse Astrocytoma; GBM, Glioblastoma; NA; Not Available; Gd, Gadolinium; AED, Antiepileptic drug; LEV, levetiracetam.

## Data Availability

The data presented in this study are available on request from the corresponding author.

## Supplementary Materials

The following supporting information can be downloaded at: https://www.mdpi.com/article/10.3390/cancers14061449/s1, Figure S1: Flow diagram showing the case selection process.
