# Supplementary material for: Correlation of Intraoperative 5-ALA-Induced Fluorescence Intensity and Preoperative 11C-Methionine PET Uptake in Glioma Surgery
Source: Cancers (Basel). 2022 Mar 11;14(6):1449. doi: 10.3390/cancers14061449 (PMC8946621; doi:10.3390/cancers14061449)
Supplement: Supplementary file 1 [file cancers-14-01449-s001.zip › cancers-1607941-supplementary.pdf]

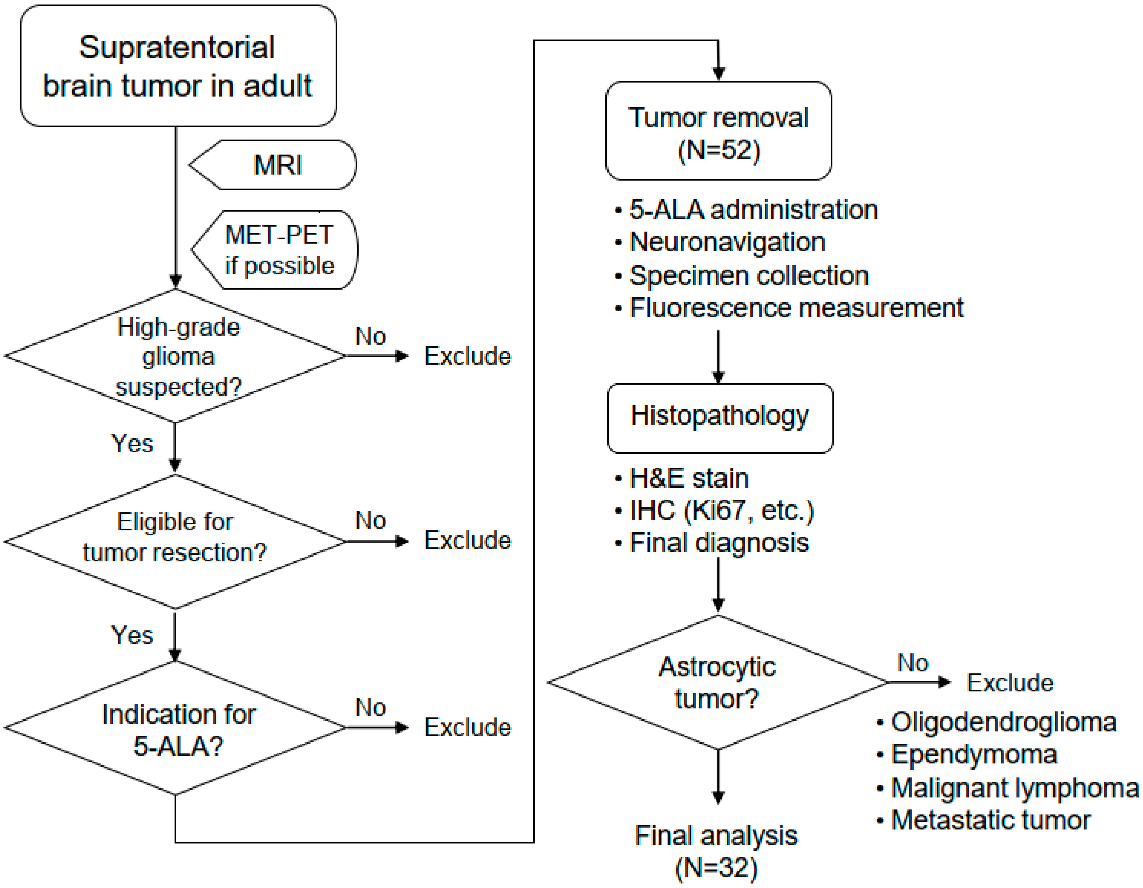

Supplementary Figure S1. Flow diagram showing the case selection process. Adult patients with suspected malignant brain tumors in supratentorial regions were subject to imaging evaluations. In those eligible for surgical resection using 5-ALA, intraoperative fluorescence intensity was measured ex vivo. The final analysis included only astrocytic tumors.
